# Patient Characteristics and Public Health Office Factors Associated With Long Reporting Delay of COVID-19 Cases in Sapporo City, Japan

**DOI:** 10.2188/jea.JE20220359

**Published:** 2024-03-05

**Authors:** Daichi Watanuki, Akiko Tamakoshi, Takashi Kimura, Toshiaki Asakura, Masayuki Saijo

**Affiliations:** 1 Department of Public Health, Graduate School of Medicine, Hokkaido University, Sapporo, Japan; 2 Department of Public Health, Faculty of Medicine, Hokkaido University, Sapporo, Japan; 3 London School of Hygiene & Tropical Medicine, University of London, London, UK; 4 Public Health Office, Health and Welfare Bureau, Sapporo Municipal Government, Sapporo, Japan

**Keywords:** COVID-19, reporting delay, patient characteristics, Public Health Office

## Abstract

**Background:**

For therapeutic efficacy, molnupiravir and nirmatrelvir-ritonavir must be started to treat patients within 5 days of disease onset to treat patients with novel coronavirus disease 2019 (COVID-19). However, some patients spend more than 5 days from disease onset before reporting to the Public Health Office. This study aimed to clarify the characteristics of patients with reporting delay.

**Methods:**

This study included data from 12,399 patients with COVID-19 who reported to the Public Health Office from March 3^rd^, 2021 to June 30^th^, 2021. Patients were stratified into “linked” (*n* = 7,814) and “unlinked” (*n* = 4,585) cases depending on whether they were linked to other patients. A long reporting delay was defined as the difference between the onset and reporting dates of 5 days or more. Univariate and multivariate analyses were performed using log-binomial regression to identify factors related to long reporting delay, and prevalence ratios with corresponding 95% confidence intervals were calculated.

**Results:**

The proportion of long reporting delay was 24.4% (1,904/7,814) and 29.3% (1,344/4,585) in linked and unlinked cases, respectively. Risks of long reporting delay among linked cases were living alone and onset on the day with a higher 7-day daily average confirmed cases or onset on weekends; whereas, risks for unlinked cases were age over 65 years, without occupation, and living alone.

**Conclusion:**

Our results suggest the necessity to establish a Public Health Office system that is less susceptible to the rapid increase in the number of patients, promotes educational activities for people with fewer social connections, and improves access to health care.

## INTRODUCTION

Novel coronavirus disease 2019 (COVID-19), caused by severe acute respiratory syndrome coronavirus 2 (SARS-CoV-2), has remained prevalent worldwide since 2022. Patients with COVID-19 may be asymptomatic or may present with symptoms ranging from mild to severe. Generally, mild symptoms include common cold symptoms, such as cough, rhinorrhea, fever, sore throat, abnormal taste and smell, and diarrhea.^1^^,^^2^ In severe cases, patients may present with sepsis or acute respiratory distress syndrome.^3^ COVID-19 is highly contagious and characterized by various symptoms; additionally, one reason it is difficult to control is that it can even be transmitted by asymptomatic individuals.^4^

The Public Health Office (PHO) of Sapporo City, which covers approximately 2 million people, awaits all COVID-19 cases through reports from medical institutions or COVID-19-associated laboratories. Until the Omicron variant of SARS-CoV-2 appeared and the number of cases skyrocketed, the PHO of Sapporo City handled all the reports from medical institutions or COVID-19-associated laboratories in Sapporo City.^5^ Once patients with COVID-19 were detected and reported to the PHO, their staff started an active epidemiological investigation of all patients as soon as possible. In this investigation, patients confirmed with COVID-19 were interviewed regarding contact with their family members and cohabitants and behavioral history up to 2 weeks prior to the onset of symptoms, so as to determine where they were infected and who infected them.^6^^,^^7^ Using this information, close contacts were also identified and recommended to undergo the SARS-CoV-2 genome reverse-transcription polymerase chain reaction (PCR) testing. Furthermore, patients identified by the PHO decided where they would receive treatment: medical institutions, COVID-19 patient-specific prepared accommodation facilities or their own homes. Even if they stayed at home during their recovery time, their health conditions were monitored daily by the PHO staff over the phone or internet.

To reduce the disease burden of COVID-19, vaccines and therapeutics are important. Previously, treatment of COVID-19 focused on steroid use and symptomatic treatment for individual symptoms,^8^ but antiviral medications, such as molnupiravir (Lagevrio) and nirmatrelvir-ritonavir (Paxlovid pack), were approved on December 24^th^, 2021 and February 10^th^, 2022, respectively. These medications have been reported to reduce the risk of progression to severe disease and hospitalization in patients with mild to moderate symptoms with risk factors for severe disease.^9^^,^^10^ Their introduction has expanded the treatment options for patients receiving home care. These medications are effective in preventing viral proliferation; however, they must be initiated within 5 days of disease onset to acquire medication efficacy.^9^^–^^12^ As of 2022 in Japan, these medications are stockpiled and distributed to medical institutions and pharmacies under regulation, which should have been registered for approval of prescriptions operated by the central government. In Sapporo, it usually takes at least 1 day to be prescribed with medication for COVID-19 patients, which means that patients must be diagnosed with COVID-19 within 4 days of disease onset to properly obtain a benefit. However, some patients could not get access to medications because more than 5 days were spent at the time of diagnosis. In addition, delay in diagnosis or reporting from the time of disease onset might contribute to disease severity and mortality^13^^–^^18^; however, the association between reporting delay and patient characteristics has not been fully investigated.

This study aimed to clarify the characteristics of patients with COVID-19 who tested positive 5 days or more after disease onset. We expected to clarify improvement points of PHO or the target population for educational activities or interventions so that oral antiviral medications, such as molnupiravir and nirmatrelvir-ritonavir, are delivered successfully within the therapeutic window.

## METHODS

### Data collection

**Figure 1.**
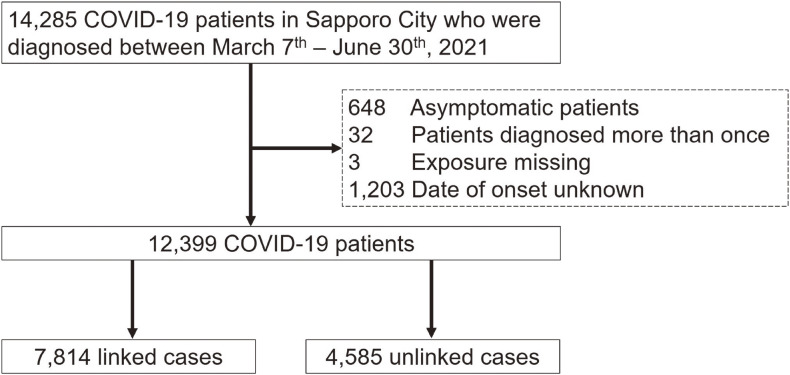
Participant flow of the present study

This study was based on data from 14,285 patients with confirmed COVID-19 from March 7^th^, 2021 to June 30^th^, 2021 and who lived in Sapporo City. The data were provided by Sapporo City PHO under an agreement between the Faculty of Medicine, Hokkaido University, and Sapporo City PHO. This period was selected to minimize the impact of differences in SARS-CoV-2 variants, COVID-19 vaccination status, and the internal PHO system to improve the efficiency of patient identification and the number of medical facilities providing COVID-19 care. During this period, the alpha variant was the main variant found in Sapporo. All patients were diagnosed with COVID-19 by confirmation using PCR test or a SARS-CoV-2 antigen detection assay. Information, such as patient age, occupation, onset date, and test-positive date, was recorded in the Sapporo City PHO database. The data on COVID-19 patients were managed only by the PHO staff from information security and privacy protection. From the dataset, 648 asymptomatic patients, 32 patients who tested positive more than once, three patients with missing exposure information, and 1,203 patients with missing onset dates were excluded. Finally, 12,399 participants were included in this study (Figure 1).

### Definition of long reporting delay

Based on the same data, the duration from disease onset to SARS-CoV-2 test-positive reporting was calculated. The disease onset was defined as the first instance wherein COVID-19 symptoms appeared. If the cases tested and resulted positive before the onset of symptoms on order of the PHO or as a screening, the duration between onset and positive confirmation may be less than 0 day. We defined “long reporting delay” as a duration of 5 days or more from disease onset to reporting.

### Potential risk factors of a long reporting delay

We considered several potential risk factors for long reporting delay, including sex, age, occupation, cohabitant information, medical history known as a risk for severity, exposure situation, the 7-day daily average confirmed cases, and the day of the week on which the first symptom appeared. Cohabitant information was categorized as living alone or with others. The medical histories included 13 diseases that were notified through office communications from the Ministry of Health, Labour and Welfare of Japan (MHLW) based on the inclusion criteria for clinical trials of the International Phase II/III Study for Molnupiravir (MOVe-OUT [002] trials), Nirmatrelvir-Ritonavir (C4671005 [EPIC-HR] trials), and the COVID-19 practice guideline.^19^^,^^20^ These were cancer, diabetes mellitus, kidney diseases, cardiovascular diseases, cerebrovascular disease, respiratory disease, smoking behavior, hypertension, obesity, hepatic disease, hematological disease, immunodeficiency, and neurological disorders. Patients were categorized according to the number of the aforementioned diseases the patients had. The 7-day daily average confirmed cases for patients were calculated from the date of onset to 6 days. We assumed that this measurement represented the burden or pressure on the PHO or medical institutions.

### Ethical consideration

The ethics committee of the Faculty of Medicine, Hokkaido University approved this study (No. 20-005).

### Statistical analysis

Cases were stratified and analyzed according to the process of PCR or antigen detection assay because the confirmation process for each patient differed depending on whether the patients were related to other COVID-19 patients. “Linked cases” were persons who were identified as having close contact with confirmed cases through active epidemiological investigation by the PHO and led to the confirmation test, while “unlinked cases” were persons who took the confirmation test without any contact information prior to disease onset or taking the test. These two groups were analyzed as separate populations because the PHO intervention had a significant impact on reporting delay. The distribution of each categorical variable is shown by linked or unlinked cases. Next, to examine the effect of each factor, we performed univariate and multivariate analyses using a log-binomial regression. The prevalence ratios (PRs) and corresponding 95% confidence intervals (CIs) were calculated. Statistical analysis was performed using SAS Enterprise Guide 7.1 (SAS Institute Inc., Cary, NC, USA).

## RESULTS

**Figure 2.**
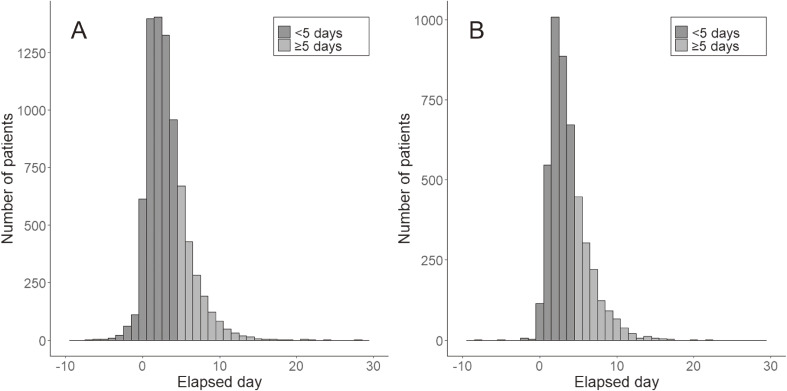
Histogram of elapsed time from disease onset to test-positive date of COVID-19 patients for (**A**) linked cases and (**B**) unlinked cases.

Among the 12,399 patients, 7,814 and 4,585 were categorized as linked and unlinked cases, respectively. The distribution of the duration from disease onset to positive test reporting is shown in Figure 2. The mean duration from onset to the test-positive reporting was 3.16 (standard deviation [SD], 2.71) day and 3.73 (SD, 2.52) day, and the proportions of cases with long reporting delay were 24.4% (1,907/7,814) and 29.3% (1,344/4,585) for linked and unlinked cases, respectively. The mean duration and proportion of cases with long reporting delays were significantly different between the linked and unlinked cases (*P* < 0.001 for each). Table 1 shows the demographic characteristics of the patients according to linked and unlinked cases. Compared to unlinked cases, linked cases were more likely to be female, aged under 18 or over 65 years, students, healthcare workers, or without occupation, and were more unlikely to be living alone.

**Table 1.** Demographics and characteristics of patients

Characteristic	Total (*N* = 12,399) *N* (%)	Linked Case (*n* = 7,814) *n* (%)	Unlinked Case (*n* = 4,585) *n* (%)
**Sex**			
Female	6,143 (49.5)	4,192 (53.6)	1,951 (42.6)
Male	6,174 (49.8)	3,598 (46.0)	2,576 (56.2)
Unknown	82 (0.7)	24 (0.3)	58 (1.3)
**Age, years**			
≤18	1,309 (10.6)	1,108 (14.2)	201 (4.4)
19–29	3,205 (25.8)	1,766 (22.6)	1,439 (31.4)
30–39	2,025 (16.3)	1,129 (14.4)	896 (19.5)
40–49	1,884 (15.2)	1,137 (14.6)	747 (16.3)
50–64	2,178 (17.6)	1,378 (17.6)	800 (17.4)
≥65	1,798 (14.5)	1,296 (16.6)	502 (10.9)
**Occupation**			
Officer or manager	864 (7.0)	525 (6.7)	339 (7.4)
Services (restaurant · pub)	913 (7.4)	513 (6.6)	400 (8.7)
Services (other)	821 (6.6)	451 (5.8)	370 (8.1)
Transportation	319 (2.6)	190 (2.4)	129 (2.8)
Students	1,569 (12.7)	1,116 (14.3)	453 (9.9)
Construction	520 (4.2)	310 (4.0)	210 (4.6)
Profession (healthcare worker)	970 (7.8)	737 (9.4)	233 (5.1)
Profession (non-health care worker)	484 (3.9)	290 (3.7)	194 (4.2)
Agricultural, Forestry, Fishing industries	12 (0.1)	7 (0.1)	5 (0.1)
Sales	457 (3.7)	236 (3.0)	221 (4.8)
Security job	55 (0.4)	35 (0.4)	20 (0.4)
Other	2,469 (19.9)	1,478 (18.9)	991 (21.6)
Without occupation	1,250 (10.1)	843 (10.8)	407 (8.9)
Unknown	1,696 (13.7)	1,083 (13.9)	613 (13.4)
**Cohabitant information**			
Living alone	2,876 (23.2)	1,416 (18.1)	1,460 (31.8)
Living with others	8,994 (72.5)	6,063 (77.6)	2,931 (63.9)
Unknown	529 (4.3)	335 (4.3)	194 (4.2)
**Medical history**			
0	7,296 (58.8)	4,705 (60.2)	2,591 (56.5)
1	3,772 (30.4)	2,281 (29.2)	1,491 (32.5)
≥2	1,331 (10.7)	828 (10.6)	503 (11.0)
**Exposure situation**			
Workplace	1,292 (10.4)	1,292 (16.5)	
Family · Corhabitant	3,376 (27.2)	3,376 (43.2)	
School	474 (3.8)	474 (6.1)	
Medical institution	503 (4.1)	503 (6.4)	
Nursing home	586 (4.7)	586 (7.5)	
Sports activities	80 (0.6)	80 (1.0)	
Drive	88 (0.7)	88 (1.1)	
Concert	36 (0.3)	36 (0.5)	
Dining together	895 (7.2)	895 (11.5)	
Trip · Homecoming	147 (1.2)	147 (1.9)	
Pub	298 (2.4)	298 (3.8)	
Other personal activity	39 (0.3)	39 (0.5)	
**7-day daily average confirmed cases**			
<100	3,049 (24.6)	1,882 (24.1)	1,167 (25.5)
100–199	3,051 (24.6)	1,885 (24.1)	1,166 (25.4)
200–299	2,140 (17.3)	1,332 (17.0)	808 (17.6)
≥300	4,159 (33.5)	2,715 (34.7)	1,444 (31.5)
**Onset day of the week**			
Monday	1,879 (15.2)	1,167 (14.9)	712 (15.5)
Tuesday	1,764 (14.2)	1,140 (14.6)	624 (13.6)
Wednesday	1,756 (14.2)	1,080 (13.8)	676 (14.7)
Thursday	1,784 (14.4)	1,143 (14.6)	641 (14.0)
Friday	1,741 (14.0)	1,106 (14.2)	635 (13.8)
Saturday	1,795 (14.5)	1,127 (14.4)	668 (14.6)
Sunday	1,680 (13.5)	1,051 (13.5)	629 (13.7)

Table 2 shows the PR of each factor associated with a long reporting delay, as assessed using log-binomial regression in linked cases. In a multivariate analysis adjusted for all factors, the risk factors for long reporting delay were: living alone compared to living with others (PR 1.14; 95% CI, 1.03–1.25); the 7-day daily average confirmed cases being 100–199 (PR 1.20; 95% CI, 1.07–1.35), or 300 or more (PR 1.16; 95% CI, 1.04–1.30) compared to that being under 100; and the symptom onset date being Thursday (PR 1.19; 95% CI, 1.03–1.38), Friday (PR 1.22; 95% CI, 1.05–1.42), Saturday (PR 1.22; 95% CI, 1.05–1.42), and Sunday (PR 1.17; 95% CI, 1.01–1.37) compared to that being on a Wednesday. On the other hand, patients aged under 18 years (PR 0.76; 95% CI, 0.65–0.89), 40–49 years (PR 0.81; 95% CI, 0.71–0.93), and 50–64 years (PR 0.86; 95% CI, 0.76–0.98) showed a lower risk for long reporting delay compared to 19–29 years. Similarly, patients with two or more medical histories showed a lower risk for long reporting delay compared to those without a medical history (PR 0.86; 95% CI, 0.73–0.997).

**Table 2.** Proportions and prevalence ratios of patients with long reporting delay for linked cases

Characteristic	Total	Long reporting delay^a^	Univariate analysis	Multivariate analysis^b^
	*N*	*n* (%)	PR (95% CI)	PR (95% CI)
**Sex**				
Female	4,192	1,006 (24.0)	Ref.	Ref.
Male	3,598	895 (24.9)	1.04 (0.96–1.12)	0.97 (0.90–1.06)
Unknown	24	6 (25.0)	1.04 (0.52–2.09)	1.05 (0.54–2.07)
**Age, years**				
≤18	1,108	247 (22.3)	0.74 (0.65–0.85)	0.76 (0.64–0.89)
19–29	1,766	529 (30.0)	Ref.	Ref.
30–39	1,129	307 (27.2)	0.91 (0.81–1.02)	0.98 (0.87–1.11)
40–49	1,137	239 (21.0)	0.70 (0.61–0.80)	0.81 (0.70–0.93)
50–64	1,378	308 (22.4)	0.75 (0.66–0.84)	0.86 (0.76–0.98)
≥65	1,296	277 (21.4)	0.71 (0.63–0.81)	0.92 (0.79–1.07)
**Occupation**				
Officer or manager	525	144 (27.4)	Ref.	Ref.
Services (restaurant, pub)	513	172 (33.5)	1.22 (1.02–1.47)	1.14 (0.93–1.39)
Services (other)	451	127 (28.2)	1.03 (0.84–1.26)	0.97 (0.79–1.18)
Transportation	190	40 (21.1)	0.77 (0.56–1.04)	0.78 (0.57–1.06)
Students	1,116	294 (26.3)	0.96 (0.81–1.14)	1.11 (0.91–1.36)
Construction	310	87 (28.1)	1.02 (0.82–1.28)	1.00 (0.79–1.25)
Profession (healthcare worker)	737	84 (11.4)	0.42 (0.33–0.53)	0.73 (0.56–0.94)
Profession (non-healthcare worker)	290	75 (25.9)	0.94 (0.74–1.20)	0.99 (0.78–1.26)
Agricultural, Forestry, Fishing industries	7	3 (42.9)	1.56 (0.66–3.72)	1.71 (0.72–4.09)
Sales	236	47 (19.9)	0.73 (0.54–0.97)	0.70 (0.53–0.94)
Security job	35	8 (22.9)	0.83 (0.45–1.56)	0.83 (0.45–1.54)
Other	1,478	368 (24.9)	0.91 (0.77–1.07)	0.93 (0.79–1.10)
Without occupation	843	221 (26.2)	0.96 (0.80–1.14)	1.15 (0.95–1.39)
Unknown	1,083	237 (21.9)	0.80 (0.67–0.95)	0.96 (0.80–1.15)
**Cohabitant information**				
Living alone	1,416	385 (27.2)	1.14 (1.03–1.25)	1.13 (1.01–1.25)
Living with others	6,063	1,451 (23.9)	Ref.	Ref.
Unknown	335	71 (21.2)	0.89 (0.72–1.09)	1.16 (0.94–1.44)
**Medical history**				
0	4,705	1,172 (24.9)	Ref.	Ref.
1	2,281	573 (25.1)	1.01 (0.92–1.10)	0.99 (0.90–1.08)
≥2	828	162 (19.6)	0.79 (0.68–0.91)	0.86 (0.73–0.997)
**Exposure situation**				
Workplace	1,292	378 (29.3)	Ref.	Ref.
Family · Corhabitant	3,376	861 (25.5)	0.87 (0.79–0.97)	0.90 (0.80–1.01)
School	474	87 (18.4)	0.63 (0.51–0.77)	0.65 (0.52–0.81)
Medical institution	503	46 (9.1)	0.31 (0.23–0.42)	0.36 (0.26–0.49)
Nursing home	586	49 (8.4)	0.29 (0.22–0.38)	0.30 (0.22–0.41)
Sports activities	80	17 (21.3)	0.73 (0.47–1.12)	0.70 (0.46–1.08)
Drive	88	27 (30.7)	1.05 (0.76–1.45)	0.92 (0.67–1.28)
Concert	36	9 (25.0)	0.85 (0.48–1.51)	0.82 (0.46–1.45)
Dining together	895	285 (31.8)	1.09 (0.96–1.24)	1.04 (0.91–1.18)
Trip · Homecoming	147	41 (27.9)	0.95 (0.73–1.25)	0.91 (0.69–1.19)
Pub	298	97 (32.6)	1.11 (0.93–1.34)	0.90 (0.72–1.11)
Other personal activity	39	10 (25.6)	0.88 (0.51–1.51)	0.87 (0.51–1.50)
**7-day daily average confirmed cases**				
<100	1,882	403 (21.4)	Ref.	Ref.
100–199	1,885	482 (25.6)	1.19 (1.06–1.34)	1.20 (1.07–1.35)
200–299	1,332	331 (24.8)	1.16 (1.02–1.32)	1.10 (0.97–1.25)
≥300	2,715	691 (25.5)	1.19 (1.07–1.32)	1.17 (1.05–1.30)
**Onset day of the week**				
Monday	1,167	288 (24.7)	1.11 (0.96–1.29)	1.14 (0.99–1.33)
Tuesday	1,140	227 (19.9)	0.90 (0.76–1.05)	0.91 (0.77–1.07)
Wednesday	1,080	240 (22.2)	Ref.	Ref.
Thursday	1,143	299 (26.2)	1.18 (1.02–1.37)	1.19 (1.03–1.38)
Friday	1,106	294 (26.6)	1.20 (1.03–1.39)	1.22 (1.05–1.42)
Saturday	1,127	292 (25.9)	1.17 (1.00–1.35)	1.23 (1.06–1.42)
Sunday	1,051	267 (25.4)	1.14 (0.98–1.33)	1.17 (1.01–1.37)

CI, confidence interval; PR, prevalence ratio.

^a^The duration between disease onset to SARS-CoV-2 test-positive reporting was 5 days or more.

^b^All factors were included in multivariate analysis.

Table 3 shows the results of the same analysis for the unlinked cases. The risks for long reporting delay were observed in patients aged 65 years or older compared to those aged 19–29 years (PR 1.23; 95% CI, 1.04–1.45); patients whose occupation was related to services (restaurants or pub) (PR 1.26; 95% CI, 1.01–1.57) or without occupation (PR 1.42; 95% CI, 1.14–1.77) compared to officers or managers; patients who were living alone compared to those living with others (PR 1.15; 95% CI, 1.05–1.26); and when the symptom onset day was Friday (PR 1.22; 95% CI, 1.04–1.44) compared to that being on Wednesday.

**Table 3.** Proportions and prevalence ratios of patients with long reporting delay for unlinked cases

Characteristic	Total	Long reporting delay^a^	Univariate analysis	Multivariate analysis^b^
	*N*	*n* (%)	PR (95% CI)	PR (95% CI)
**Sex**				
Female	1,951	602 (30.9)	Ref.	Ref.
Male	2,576	728 (28.3)	0.92 (0.84–1.00)	0.94 (0.86–1.03)
Unknown	58	14 (24.1)	0.78 (0.49–1.24)	0.79 (0.50–1.26)
**Age, years**				
≤18	201	50 (24.9)	0.90 (0.70–1.16)	0.90 (0.68–1.19)
19–29	1,439	399 (27.7)	Ref.	Ref.
30–39	896	267 (29.8)	1.07 (0.94–1.22)	1.11 (0.97–1.27)
40–49	747	211 (28.2)	1.02 (0.88–1.17)	1.07 (0.92–1.23)
50–64	800	234 (29.3)	1.05 (0.92–1.21)	1.14 (0.99–1.32)
≥65	502	183 (36.5)	1.31 (1.14–1.52)	1.23 (1.04–1.45)
**Occupation**				
Officer or manager	339	93 (27.4)	Ref.	Ref.
Services (restaurant, pub)	400	136 (34.0)	1.24 (0.99–1.55)	1.25 (1.01–1.56)
Services (other)	370	108 (29.2)	1.06 (0.84–1.35)	1.08 (0.85–1.37)
Transportation	129	36 (27.9)	1.02 (0.73–1.41)	1.04 (0.75–1.44)
Students	453	124 (27.4)	1.00 (0.79–1.25)	1.10 (0.85–1.42)
Construction	210	59 (28.1)	1.02 (0.78–1.35)	1.04 (0.79–1.38)
Profession (healthcare worker)	233	45 (19.3)	0.70 (0.51–0.96)	0.68 (0.50–0.93)
Profession (non-healthcare worker)	194	44 (22.7)	0.83 (0.61–1.13)	0.83 (0.61–1.13)
Agricultural, Forestry, Fishing industries	5	2 (40.0)	1.46 (0.49–4.33)	1.37 (0.46–4.05)
Sales	221	60 (27.1)	0.99 (0.75–1.31)	1.02 (0.77–1.35)
Security job	20	3 (15.0)	0.55 (0.19–1.57)	0.59 (0.21–1.70)
Other	991	262 (26.4)	0.96 (0.79–1.18)	0.97 (0.79–1.19)
Without occupation	407	163 (40.0)	1.46 (1.18–1.80)	1.43 (1.15–1.78)
Unknown	613	209 (34.1)	1.24 (1.01–1.53)	1.18 (0.95–1.46)
**Cohabitant information**				
Living alone	1,460	465 (31.9)	1.15 (1.05–1.26)	1.14 (1.04–1.25)
Living with others	2,931	812 (27.7)	Ref.	Ref.
Unknown	194	67 (34.5)	1.24 (1.02–1.53)	1.13 (0.92–1.40)
**Medical history**				
0	2,591	770 (29.7)	Ref.	Ref.
1	1,491	429 (28.8)	0.97 (0.88–1.07)	0.93 (0.84–1.03)
≥2	503	145 (28.8)	0.97 (0.84–1.13)	0.87 (0.75–1.02)
**7-day daily average confirmed cases**				
<100	1,167	371 (31.8)	Ref.	Ref.
100–199	1,166	342 (29.3)	0.92 (0.82–1.04)	0.95 (0.84–1.08)
200–299	808	209 (25.9)	0.81 (0.70–0.94)	0.83 (0.71–0.96)
≥300	1,444	422 (29.2)	0.92 (0.82–1.03)	0.92 (0.82–1.03)
**Onset day of the week**				
Monday	712	203 (28.5)	1.01 (0.85–1.19)	1.05 (0.89–1.24)
Tuesday	624	174 (27.9)	0.99 (0.83–1.17)	1.02 (0.85–1.21)
Wednesday	676	191 (28.3)	Ref.	Ref.
Thursday	641	195 (30.4)	1.08 (0.91–1.27)	1.09 (0.92–1.28)
Friday	635	211 (33.2)	1.18 (1.00–1.38)	1.22 (1.04–1.44)
Saturday	668	202 (30.2)	1.07 (0.91–1.26)	1.13 (0.96–1.34)
Sunday	629	168 (26.7)	0.95 (0.79–1.13)	0.99 (0.83–1.18)

CI, confidence interval; PR, prevalence ratio.

^a^The duration between disease onset to SARS-CoV-2 test-positive reporting was 5 days or more.

^b^All factors were included in multivariate analysis.

## DISCUSSION

This study confirmed that the risk factors for a long reporting delay differed between linked and unlinked cases. Regarding patient characteristics, patients with long reporting delay among linked cases tended to age 19–29 years, living alone, and without medical history; while patients with long reporting delay among unlinked cases tended to be aged older than 65 years, without occupation, and living alone. Regarding PHO operations, the onset day of the week and the number of daily confirmed cases were associated with long reporting delay among the linked cases.

Previous studies have also found that patients who were identified through active epidemiological investigation (ie, linked cases) have shorter durations of onset to reporting than unlinked cases.^21^^–^^23^ The initial symptoms of COVID-19 are usually mild and do not occur suddenly; thus, it might be difficult for patients to recognize early symptoms of the disease. Moreover, because the symptoms differ between individuals, it is possible that patients may not receive SARS-CoV-2 testing in a timely manner. However, the authors pointed out that such a situation can be avoided by testing and health observation by PHO staff through information gleaned from already confirmed cases through active epidemiological investigation.^21^ For these reasons, linked cases might show a lower proportion of long reporting delay than unlinked cases. In a previous study in which long reporting delay was defined as 6 days or more, the risk of long reporting delay was significantly higher in unlinked cases than in linked cases.^22^ In another study that described clinical time delay distributions, the mean duration of reporting delay among linked cases was 2.96 (95% CI, 2.95–2.98) days and was shorter than that among unlinked cases (mean 3.31; 95% CI, 3.30–3.32 days). Also, the duration of unlinked cases was significantly longer than that of linked cases.^23^ A previous study conducted in Japan reported that the long reporting delay among unlinked cases was due to the policy issued by the National Government, which directed the patients to wait for a consultation with a PHO or visit a physician within 4 days of disease onset.^22^ Although such criteria were already withdrawn by the National Government during the study period, similar results were obtained in this study. This may be because of the policy issued by the National Government, which remained to some degree, after withdrawal of the policy.

According to the results of this study, linked patients aged under 18, 40–49, and 50–64 years showed a significantly lower risk for long reporting delay compared to 19–29 years. In other words, patients aged 19–29 years may be at risk of long reporting delays compared to other age groups. Similarly, linked patients without a medical history were at risk of a long reporting delay compared to patients with two or more medical histories. Patients who were 19–29 years old or without any medical history might possibly be reluctant to undergo the SARS-CoV-2 test because they often have milder symptoms than the other age groups.^24^ Moreover, these patients were considered to be at lower risk of developing severe conditions, which might have affected the priority of response at the PHO. In contrast, children tend to have milder symptoms,^25^ but the proportion of long reporting delays was lower in those under 18 years of age. In addition to the presence of parents who support medical examinations, Sapporo City has a medical subsidy system for children.^26^ This further lowers the hurdle for them to receive medical care and is thought to be the reason why children are more likely to undergo early medical examinations.

In the results of the linked cases, the 7-day daily average confirmed cases and the day of the week at which there was onset of symptoms, were also associated with long reporting delay; both of which were related to the operation of the PHO. The PHO system is dependent on human power in all epidemiological investigations. According to this system, if the number of positive cases increase rapidly, it would take more days from the epidemiological investigation to the coordination of PCR testing and its consequent diagnosis, which may have led to a delay in reporting. As for the day of onset being on weekends, the availability of medical institutions or laboratories to receive confirmation testing was limited. Moreover, recording the test results at the PHO may take more time, even if testing itself was coordinated as usual. Therefore, the burden on PHO operations caused by their tasks depending on human power might be one of the factors for a long reporting delay. In contrast to linked cases, unlinked cases were required to take action to receive medical examinations themselves. Thus, they may be less likely to be affected by PHO operation. However, the lower 7-day daily average confirmed cases and onset on Fridays were related to long reporting delays among unlinked cases. The reasons are not clear; however, if the number of infected patients in the city is small, which means there is little chance of contact with infected patients, subjects may be unlikely to suspect COVID-19 infection. In cases with onset on Fridays, as the weekend approaches, many people are likely to wait for the beginning of the next week and check their condition even if they have some symptoms.

Living alone was a risk factor for long reporting delay in both linked and unlinked cases. People must undergo SARS-CoV-2 testing when they have symptoms, such as fever and respiratory symptoms. However, patients with suspected COVID-19 are strictly prohibited from using public transportation in Japan. Therefore, they need to reach a medical institution that provides SARS-CoV-2 testing by themselves, with assistance from others, or wait for the PHO’s arrangement of transportation or to receive a test kit delivered by the PHO. For these reasons, it is possible that those living alone had difficulties and required more time to undergo PCR tests.

In addition to living alone, age and occupation status were found to be risk factors for a long reporting delay among unlinked cases. Patients with infectious diseases, such as COVID-19, need to undergo medical tests, be transported, and receive medical care; these require support from the surrounding community including family members, cohabitants, supportive friends, or neighbors. Patients who are old, without occupation, or living alone might have less frequent contact with others in their closest proximity, and they have fewer opportunities to receive advice or information necessary for support; thus, they are less likely to undergo medical tests in a timely manner.

Earlier administration of antiviral drugs is expected to be more effective in reducing the severity and mortality of acute viral infections, such as COVID-19, and improving the reporting delay may contribute to their reduction. To shorten the reporting delay, people suspected of having COVID-19 with the risk factors mentioned above should be aware of the way they undergo medical tests. Simultaneously, improving the capacity of medical tests and amending access to medical care that does not depend on public transportation or emergency medical services are important to facilitate medical test-seeking behavior. In addition, our study found that factors affected by the operation of the PHO were associated with long reporting delay; therefore, it is necessary to proactively utilize Information Technology to automate and simplify patient registration, and information sharing will also help reduce the burden on PHO or medical institution.

The strength of this study is the use of information on all patients diagnosed in one region of Japan. More detailed patient information compared with previous studies allowed us to analyze the characteristics of patients with long reporting delay and the factors of operating systems in the PHO contributing to a long reporting delay. This study had some limitations. We only used data from the fourth wave of COVID-19 in Sapporo. During this period, the alpha variant was the predominant type, and vaccination was not sufficiently widespread; thus, the impact of current variants, vaccination, or the development of an internal PHO system cannot be mentioned.

In conclusion, this study revealed that factors associated with long reporting delays differed between linked and unlinked cases. The study suggested that it is necessary to establish a PHO system that does not depend on human power to handle a rapid increase in the number of COVID-positive cases and is less susceptible to the impact of onset days of the week. Improved healthcare access and educational activities, especially for people with factors that tend to reduce social connections, such as old age, lack of occupation, and living alone, are also necessary.
